# Diagnostic utility of monitoring cytomegalovirus-specific immunity by QuantiFERON-cytomegalovirus assay in kidney transplant recipients

**DOI:** 10.1186/s12879-018-3075-z

**Published:** 2018-04-16

**Authors:** Dominika Deborska-Materkowska, Agnieszka Perkowska-Ptasinska, Anna Sadowska, Jolanta Gozdowska, Michał Ciszek, Marta Serwanska-Swietek, Piotr Domagala, Dorota Miszewska-Szyszkowska, Elzbieta Sitarek, Agnieszka Jozwik, Artur Kwiatkowski, Magdalena Durlik

**Affiliations:** 1 Department of Transplantation Medicine, Nephrology, Internal Diseases, T. Orłowski Institute of Transplantation Medical University of Warsaw, 59 Nowogrodzka Street, 02-006 Warsaw, Poland; 2 Department of Immunology, Transplantology, Internal Diseases, T. Orłowski Institute of Transplantation Medical University of Warsaw, 59 Nowogrodzka Street, 02-006 Warsaw, Poland; 3 Department of General and Transplant Surgery, T. Orłowski Institute of Transplantation Medical University of Warsaw, 59 Nowogrodzka Street, 02-006 Warsaw, Poland

**Keywords:** Kidney transplantation, QuantiFERON-cytomegalovirus, Postprophylaxis, Cytomegalovirus infection, Hypogammaglobulinemia, Valganciclovir

## Abstract

**Background:**

Despite universal prophylaxis, late cytomegalovirus (CMV) infection occurs in a high proportion of kidney transplant recipients. We evaluated whether a specific viral T-cell response allows for the better identification of recipients who are at high risk of CMV infection after prophylaxis withdrawal.

**Methods:**

We conducted a prospective study in 19 pretransplant anti-CMV seronegative kidney graft recipients R- (18 from seropositive donors [D+] and one from a seronegative donor [D-]) and 67 seropositive recipients R(+) (59 from seropositive donors and eight from seronegative donors) who received antiviral prophylaxis with valganciclovir. The QuantiFERON-CMV (QF-CMV) assay was performed within the first and third months after transplantation. Blood samples were monitored for CMV DNAemia using a commercial quantitative nucleic acid amplification test (QNAT) that was calibrated to the World Health Organization International Standard.

**Results:**

Twenty-one of the 86 patients (24%) developed CMV viremia after prophylaxis withdrawal within 12 months posttransplantation. In the CMV R(+) group, the QF-CMV assay yielded reactive results (QF-CMV[+]) in 51 of 67 patients (76%) compared with 7 of 19 patients (37%) in the CMV R(−) group (*p* = 0.001). In the CMV R(+) group, infection occurred in seven of 16 recipients (44%) who were QF-CMV(−) and eight of 51 recipients (16%) who were QF-CMV(+). In the CMV R(−) group, infection evolved in five of 12 recipients (42%) who were QF-CMV(−) and one of 7 recipients (14%) who were QF-CMV(+). No difference was found in the incidence of CMV infection stratified according to the QF-CMV results with regard to the recipients’ pretransplant CMV IgG serology (*p* = 0.985). Cytomegalovirus infection occurred in 15 of 36 patients (42%) with hypogammaglobulinemia (HGG) 90 days posttransplantation compared with two of 34 patients (6%) without HGG (*p* = 0.0004). Cytomegalovirus infection occurred in seven of 13 patients (54%) with lymphocytopenia compared with 14 of 70 patients (20%) without lymphocytopenia (*p* = 0.015). The multivariate analysis revealed that the nonreactive QuantiFERON-CMV assay was an independent risk factor for postprophylaxis CMV infection.

**Conclusions:**

In kidney transplant recipients who received posttransplantation prophylaxis, negative QF-CMV results better defined the risk of CMV infection than initial CMV IgG status after prophylaxis withdrawal. Hypogammaglobulinemia and lymphocytopenia were risk factors for CMV infection.

## Background

Despite remarkable advances in the diagnostic and therapeutic modalities for its management, cytomegalovirus (CMV) remains a significant cause of serious infectious complications and occasionally mortality in immunocompromised patients [1]. Solid organ transplant recipients are at high risk for CMV infection, especially during the first 3–12 months after transplantation, because of high initial immunosuppression. Two main strategies are used to prevent CMV infection: prophylaxis of viral infections using antiviral drugs and preemptive therapy for organ recipients who develop evidence of CMV infection during routine screening [2–4]. Both strategies have resulted in significant reductions of CMV infection and CMV-related mortality. Prophylaxis usually begins shortly after transplantation and continues for a finite period of time, often in the range of 3–6 months. However, such a strategy has not led to the elimination of postprophylaxis CMV infection. Moreover, this strategy has led to a higher risk of developing anti-CMV drug resistance, a higher cost of antiviral medications, and a greater risk of side effects, with many patients who are overtreated [5, 6].

The highest risk of CMV disease involves 15–25% of organ transplant recipients who are seronegative for CMV (R-) and receive organs from seropositive donors (D+) [7]. Thus, the management of CMV infection in kidney graft recipients has been driven by donor and recipient pretransplant serology [8, 9]. However, the incidence of CMV infection is significant not only in high-risk recipients (D+/R-) but also in lower-risk recipients (D−/R+, D+/R+) [4, 10–12]. Moreover, some high-risk patients never develop CMV infection despite not receiving any prophylaxis treatment [2, 13]. These observations suggest that both the presence of CMV-specific IgG antibodies and prolonged immune system activation contribute to the development of CMV infection after transplantation.

We evaluated whether a specific viral T-cell response allows for the better identification of recipients who are at high risk of CMV infection after prophylaxis withdrawal.

## Methods

### Study design and participants

**Fig. 1:**
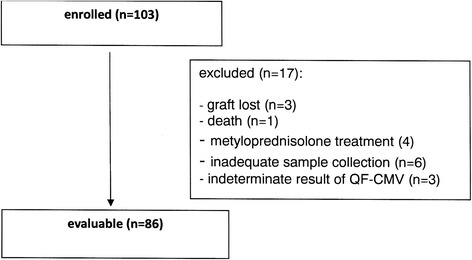
Baseline characteristics of the study participants

We conducted a prospective follow-up study of kidney graft transplant recipients in the Department of General and Transplant Surgery, T. Orłowski Institute. A total of 103 consecutive adult kidney transplant recipients were initially enrolled in the study between April and November 2014. To make the study population more homogeneous, we excluded 17 recipients from the analysis (Fig. 1).

The remaining 86 kidney transplant recipients were enrolled in the study and were followed for 360 days posttransplantation. According to the institutional protocol, all but one patient (D−/R-) received antiviral prophylaxis with valganciclovir, with the dose adjusted to kidney graft function. CMV prophylaxis was given for 3 months in all but five patients. CMV prophylaxis was stopped before the end of the third month in these recipients because of severe leucopenia that did not respond to mycophenolate mofetil dose reduction and/or GM*-*CSF treatment.

Immunosuppression was performed according to the institutional protocol. A total of 80.2% of the recipients received induction therapy, and 98.0% received triple maintenance therapy with a combination of prednisone, tacrolimus, and antimetabolite (mycophenolate mofetil or mycophenolate sodium; Table 1).

**Table 1:** Baseline characteristics of the study patients

Characteristics	
Age of recipient, years	Median: 46.5 (range: 20–74)
Gender (male) N (%)	57 (66%)
Type of transplant N (%)	
Kidney	82 (95.4%)
Kidney + pancreas	4 (4.6%)
Type of donor N (%)	
Living	8 (9.3%)
Deceased	78 (90.7%)
Pretransplant donor (D)/recipient (R) CMV serostatus N (%)	
D+/R-	18 (20.9%)
D−/R-	1 (1.1%)
D−/R+	8 (9.4%)
D+/R+	59 (68.6%)
Induction therapy N (%)	
Thymoglobulin	7 (8.1%)
Basiliximab	62 (72.1%)
None	17 (19.8%)
Maintenance immunosuppression N (%)	
Tacrolimus +mycophenolate mofetil + prednisone	77 (89.5%)
Tacrolimus + mycophenolate sodium + prednisone	7 (8.1%)
Cyclosprine A + mycophenolate mofetil + prednisone	1 (1.2%)
Tacrolimus + everolimus + prednisone	1 (1.2%)
The time of VGCV initiation (days), mean ± SD [range]	9 ± 4.7 [0–25]
The time of VGCV discontinuation (days), mean ± SD [range]	92.4 ± 13.1 [53–122]
Duration of antiviral prophylaxis (days), mean ± SD [range]	83.1 ± 13.4 [40–113]
Tacrolimus concentration (ng/mL), mean ± SD [range]	
Day 30	10.4 ± 3.7 [0.3–23]
Day 90	9.5 ± 4.5 [0.3–25.2]
Day 360	7.4 ± 2.8 [3.2–22.4]
Allograft function (eGFR; mL/min/1.73 m2), mean ± SD [range]	
Day 30	48.7 ± 21.9 [6.7–103.7]
Day 90	50.4 ± 21.0 [8.5–98.9]
Day 360	51.3 ± 20.4 [10.2–96.7]

*VGCV* valganciclovir, *eGFR* estimated glomerular filtration rate

The primary endpoint of the study was the incidence of CMV infection within 360 days posttransplantation. We hypothesized that the CMV-specific T-cell assay that was performed early posttransplantation may allow for the better identification of recipients who are at risk of postprophylaxis CMV infection. The secondary objective was the analysis of other potential risk factors for postprophylaxis CMV infection. Based on the criteria recommended by the American Society of Transplantation for use in clinical trials, the following definitions applied:

- CMV infection: CMV DNAemia regardless of symptoms.
- CMV disease: evidence of CMV infection with attributable symptoms.
- CMV DNAemia without symptoms: detection of CMV DNA in plasma [8].

#### Procedures

Cytomegalovirus DNA quantification was performed using plasma samples on days 30, 90, and 360 post-kidney transplantation and additionally at the discretion of the treating physician. Cytomegalovirus DNAemia was evaluated in plasma using a commercial quantitative nucleic acid amplification test (SmartCycler II, Cepheid AB, Sunnyvale, CA, USA) that was calibrated to the 1st World Health Organization International Standard. The limit of detection was 50 copies/ml, with linearity of 500–107 copies/ml. The CMV-specific T-cell response was evaluated at three time points: 7, 30, and 90 days posttransplantation.

##### QuantiFERON-CMV analysis

QuantiFERON-CMV is an enzyme-linked immunosorbent assay-based interferon-γ (IFN-γ) release CD8+ assay that has been accepted and commercialized by the European Union. The assay was performed according to the manufacturer’s instructions. Peripheral blood was collected in three (3 × 1 ml) heparinized tubes. Tube 1 contained a mixture of human cytomegalovirus peptide epitopes. Tube 2 contained phytohemagglutinin (positive control). Tube 3 contained sterile phosphate-buffered saline (negative control). After blood collection by venipuncture, the tubes were gently shaken (10 times up and down) and then incubated overnight for 18–24 h at 37 °C. All of the tubes were then centrifuged for 15 min at 2500 relative centrifugal force, and plasma was collected and stored at − 80 °C. Specific IFN-γ levels were measured using a standard enzyme-linked immunosorbent assay. According to the manufacturer, the results were considered positive when the peptide response was ≥0.2 IU/ml of IFN-γ. If the level of IFN-γ in the CMV antigen tube was < 0.1 IU/mL and in the mitogen tube was < 0.5 IU/mL, then the result was considered indeterminate [14].

##### Detection of CMV-specific antibodies

Specific serum anti-CMV IgG titer was measured with the ARCHITECT CMV IgG assay (Abbott Laboratories, Dublin, Ireland) according to the manufacturer’s instructions. The ARCHITECT CMV IgG assay is a chemiluminescent microparticle immunoassay that is designed to have a precision of ≤10% total (total is an accumulation of within run, between run and between day) CV (coefficient of variation) for representative specimens within the ranges of 6–60 AU/ml and 200–250 AU/ml. Among transplant recipients, relative sensitivity is 100% (lower 95% confidence limit of 91.96%) and specificity is 100% (lower 95% confidence limit of 93.62%; from the manufacturer’s information brochure). Other commercial tests were performed according to the manufacturer’s instructions.

Hypogammaglobulinemia (HGG) was defined as a total gamma globulin blood concentration < 0.8 g/dl, determined by capillary electrophoresis. Gammaglobulin blood concentration was examined on day 0 (before transplantation) and on day 90 post-kidney transplantation. Lymphocytopenia was defined as a lymphocyte blood count < 0.8 G/L, determined by fluorescence flow cytometry. Lymphocyte blood count was evaluated at three time points: 0, 30, and 90 days posttransplantation. The estimated glomerular filtration rate was calculated by the abbreviated Modification of Diet in Renal Disease equation.

### Ethics

The study was approved by the Ethical Committee of Medical University of Warsaw and complied with the provisions of the Good Clinical Practice Guidelines and the Declaration of Helsinki. All patients provided written informed consent prior to participation.

### Statistical analyses

Qualitative variables were compared using the *χ*^*2*^ statistic that follows the *χ*^*2*^ distribution (*χ*^*2*^ test) or hypergeometric distribution (Fisher’s exact test), depending on the sample size (i.e., *χ*^*2*^ test when the expected values are > 5 and Fisher’s exact test otherwise). The Mantel-Haenszel test was used to analyze the odds for stratified sampled data. The test is based on summary measures of associations and provides a weighted average of the odds ratio (OR). Quantitative variables were summarized as medians (ranges) because the parameters did not follow a normal distribution and were compared using the Wilcoxon rank-sum test. Multidimensional analysis was performed using generalized logistic regression models (GLMs). To assess the goodness-of-fit and select the optimal model, the Akaike information criterion was used. Values of *p* < 0.05 were considered statistically significant. The data were analyzed using the SAS 13.2 software.

## Results

### Patient characteristics

The pretransplant D/R serostatus and baseline characteristics of the recipients are summarized in Table 1.

### Postprophylaxis CMV infection

No episode of CMV infection was observed while the patients were on antiviral prophylaxis. Twenty-one of 86 patients developed postprophylaxis CMV infection within 12 months posttransplantation (Table 2).

**Table 2:** Incidence of CMV infection according to donor (D) and recipient (R) pretransplant serostatus

D/R (N)	No CMV infection (N)	CMV infection	
		CMV DNAemia without symptoms (N)	CMV disease (N)
D+/R- 18	12	1	5
D+/R+ 59	47	2	10
D−/R+ 8	5	0	3
D−/R- 1	1	0	0

Cytomegalovirus infection occurred at a median of 48 days after the discontinuation of antiviral prophylaxis. Among the 18 patients with CMV disease, defined as evidence of CMV infection with attributable symptoms, the QuantiFERON-CMV analysis yielded positive results in 10 recipients (56%). All of the patients with DNAemia without clinically symptomatic CMV disease had negative QuantiFERON-CMV. The proportions of CMV infection by covariate are shown in Table 3.

**Table 3:** Relationship between selected parameters and occurrence of posttransplant CMV infection

Characteristics	CMV infection	No CMV infection	p
	*N* = 21	*N* = 65	
Age, mean ± SD, y	51.4 ± 13.8	46.8 ± 15.6	0.244
Gender, male (%)	57.1	69.2	0.308
Type of transplant, Kidney /Kidney + pancreas (%)	24.4/25.0	75.6/75.0	0.977
Allograft function, (eGFR), mean ± SD			
Day 30	44.0 ± 15.4	50.2 ± 23.3	0.347
Day 90	43.7 ± 17.4	52.6 ± 21.8	0.136
Blood transfusion, N Yes/No	12 /9	24 /38	0.140
Donor, D+/D- (%)	23.4/33.3	76.6/66.7	0.682
Pretransplant recipient CMV serostatus R+/R- (%)	22.4/33.1	77.6/66.9	0.410
The time of VGCV initiation after KTx (days); mean ± SD	9 ± 5	9 ± 5	0.905
The time of VGCV discontinuation after KTx (days); mean ± SD	94 ± 9	92 ± 14	0.458
Duration of antiviral prophylaxis (days); mean ± SD	85 ± 9	82 ± 15	0.382
total IgG concentration (g/L) < 7 / ≥7%			
Day 0	0.0/26.3	100.0/73.7	0.571
Day 90	41.1/18.0	58.9/82.0	0.057
anti-CMV IgG titer (AU/ml) mean ± SD			
Day 0	138 ± 113	168 ± 109	0.803
Day 90	113 ± 85	159 ± 109	0.087
leukocyte blood count (G/L) mean ± SD			
Day 0	7.8 ± 2.4	7.9 ± 2.3	1.000
Day 30	8.5 ± 2.7	9.2 ± 4.0	0.800
Day 90	6.6 ± 4.9	6.5 ± 3.2	0.080
Induction therapy (%)			0.026
Yes basiliximab	25.8	74.2	
Yes thymoglobulin	57.1	42.9	
None	5.9	94.1	
QuantiFERON-CMV assay (IU/mL) < 0.2/≥0.2 (%)			
All time points used	42.9/15.6	57.1/84.4	0.005
Day 7	39.0/14.6	61.0/85.4	0.015
Day 30	40.7/9.1	59.3/90.9	0.003
Day 90	42.8/16.4	57.2/83.6	0.008
lymphocyte blood count (G/L) < 0.8/≥0.8 (%)			
Day 0	0.0/25.3	100.0/74.7	1.000
Day 30	42.9/21.0	57.1/79.0	0.090
Day 90	53.8/20.0	46.2/80.0	0.015
gammaglobulin blood concentration (g/dL) < 0.8/≥0.8 (%)			
Day 0	18.2/24.8	81.8/75.2	0.623
Day 90	41.7/5.9	58.3/94.1	0.0004

#### Risk factors of postprophylaxis CMV infection

##### Serology and QuantiFERON-CMV analysis - relationship with postprophylaxis CMV infection

The QuantiFERON-CMV assay was performed at all three time-points in 71 patients (83%) and at two time-points in 15 patients (17%). Among the 67 CMV R+ patients, the QuantiFERON-CMV analysis yielded positive results in 51 recipients (76%) compared with seven recipients (37%) in the CMV R- group (*p* = 0.001). We assessed the value of the QuantiFERON-CMV assay for predicting protection against CMV infection at three time-points. At each time-point, patients with positive QuantiFERON-CMV had a significantly lower subsequent incidence of CMV infection than patients with a nonreactive assay (Table 3).

**Fig. 2:**
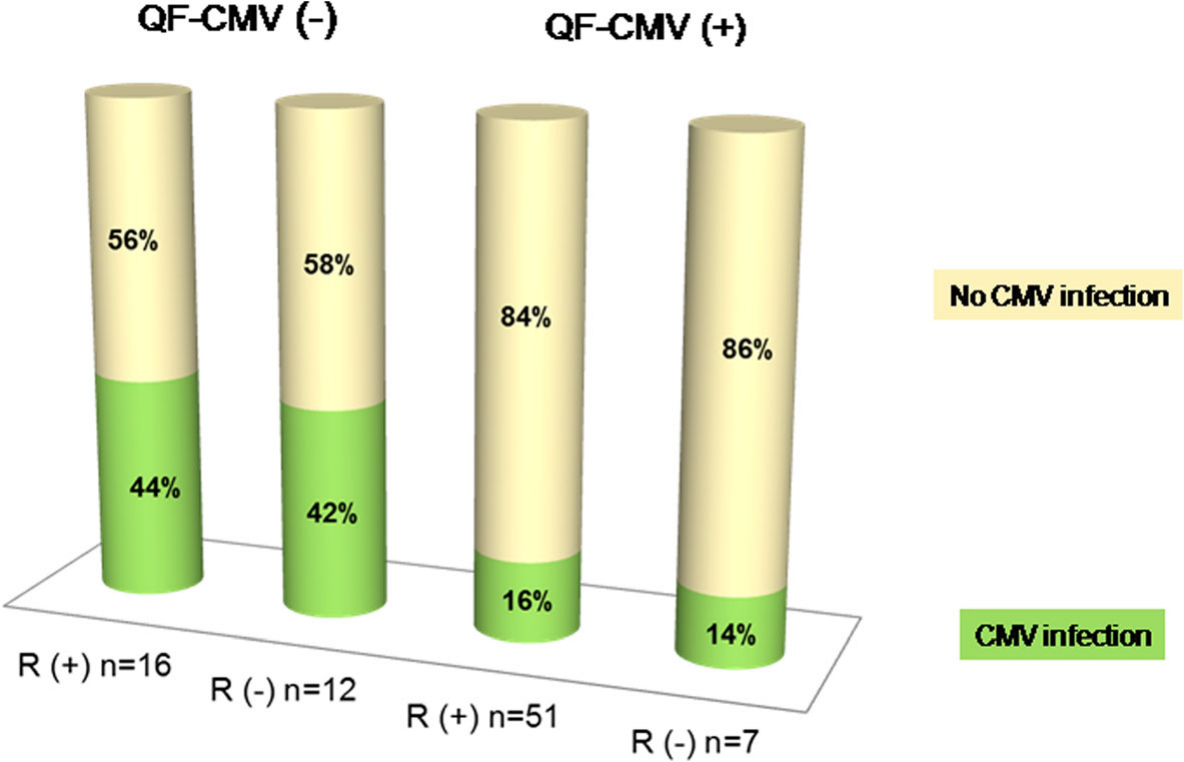
Incidence of CMV infection stratified according to QuantiFERON-CMV with regard to the recipient’s pretransplant CMV IgG serology

**Fig. 3:**
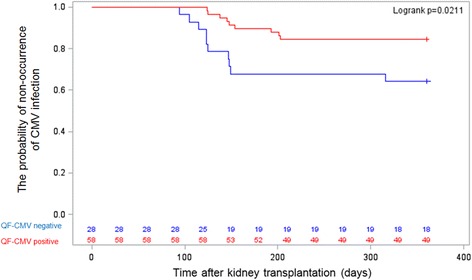
Time to development of CMV infection in patients with a positive (red line) vs. negative (blue line) QuantiFERON-CMV assay result.  QF-CMV negative,  QF-CMV positive

Cytomegalovirus infection occurred in 15 of 67 R+ recipients (22%) and six of 19 R- recipients (32%), indicating that the proportion of patients with CMV infection was comparable between groups, regardless of serological status (OR = 1.6, 95% confidence interval [Cl] = 0.51–4.92, *p* = 0.410) at the time of transplantation. Only nine of 58 recipients (16%) with cell-mediated immunity developed CMV infection compared with 12 of 28 recipients (43%) with a nonreactive QuantiFERON assay (*p* = 0.005). As shown in Fig. 2, stratified analysis using the Mantel-Haenszel method revealed no significant differences in the incidence of CMV infection stratified according to QuantiFERON-CMV assay results with regard to the recipient’s pretransplant CMV IgG serology (OR = 1.09 for CMV IgG(+) group vs. CMV IgG(−) group 95% Cl = 0.31–3.84, *p* = 0.985). The incidence of CMV infection was significantly higher in the QuantiFERON-CMV-negative group during follow-up (Fig. 3). A Kaplan Meier curve of the time to CMV infection in recipients who were QuantiFERON-CMV-positive vs. those who were QuantiFERON-negative is shown in Fig. 3.

##### Hypogammaglobulinemia and lymphocytpenia - relationship with postprophylaxis CMV infection

Cytomegalovirus infection occurred in 15 of 36 patients (42%) with HGG on day 90 posttransplantation compared with two of 34 patients (6%) without HGG (*p* = 0.0004). Cytomegalovirus infection occurred in seven of 13 patients (54%) with lymphocytopenia compared with 14 of 70 patients (20%) who were nonlymphocytopenic (*p* = 0.015).

The multivariate analysis included all parameters that were analyzed in the univariate analyses. The maximum model to be considered was identified. The optimal subset of variables was then selected, and their reliability was assessed. The factors that were found to be independent risk factors for postprophylaxis CMV infection were HGG on day 90 posttransplantation, a nonreactive QuantiFERON-CMV assay, and lymphocytopenia on day 90 posttransplantation (Table 4). Hypogammaglobulinemia and a nonreactive QuantiFERON-CMV assay had the strongest predictive value for the incidence of postprophylaxis CMV infection.

**Table 4:** The best subset of variables with the strongest predictive value for the incidence of posttransplant CMV infection according to the multivariate analysis

The best subset of parameters	Multivariate analysis		
	OR	95% CI	P
HGG on day 90 posttransplantation	7.6	1.7–34.1	0.008
Nonreactive QuantiFERON-CMV assay	4.2	1.1–15.6	0.033
Lymphocytopenia on day 90 posttransplantation	3.5	0.6–19.2	0.146

## Discussion

To date, QuantiFERON-CMV has not been well studied with regard to the risk of postprophylaxis CMV infection in kidney transplant recipients who are subjected to such a preventive modality, regardless of pretransplant serological status.

In our study, all of the patients (except one) were either R+ or received the kidney from a seropositive donor D(+), which made them at-risk for CMV infection after transplantation. In our study, all of the patients (except one) were either R(+) or received a kidney from a seropositive donor (D[+]), which made them at-risk for CMV infection after transplantation. In the R(−) group, all of the patients who developed CMV infection and CMV disease received the kidney from D(+). This tends to confirm the fact that the CMV sero-status of both recipients and donors’ is a very important predictor of post-transplant CMV infection and CMV disease.

However, a major finding of the present study was that a negative QuantiFERON-CMV result better defines the risk of postprophylaxis CMV infection than pretransplant CMV serostatus. Furthermore, negative QuantiFERON-CMV results appear to be even more predictive of the individual risk for CMV infection. Our results are consistent with the report of Kumar et al. [15]. These authors evaluated the predictive value of the QuantiFERON assay for determining whether patients who complete a course of therapy for CMV DNAemia require secondary antiviral prophylaxis. At the end of treatment, 48% of the recipients had a negative QuantiFERON-CMV. The recurrence of CMV infection was observed in 69% of QuantiFERON-CMV-negative recipients, despite more prolonged antivirals, in contrast to 7% of recipients who had a positive QuantiFERON-CMV (*p* = 0.001).

An increasing number of studies have been conducted to investigate the feasibility of immune monitoring of the CMV-specific T-cell response as a clinical marker for predicting CMV infection posttransplantation. Sester et al. analyzed CMV-specific T-cell levels in 96 solid organ transplant recipients. They reported a strong correlation between low absolute numbers of CMV-specific CD4 T cells and the frequency of infectious episodes in lung but not kidney transplant recipients [16]. Eid et al. analyzed IFN-γ-producing CMV-specific CD4+ and CD8+ T cells at serial time-points among 44 high- and intermediate-risk kidney transplant recipients who received 3 months of valganciclovir prophylaxis. The study found no significant association between CMV-specific T cells and the time to CMV DNAemia, but the conclusions were limited by the small number of patients who experienced CMV DNAemia [17]. Nonetheless, the relatively homogeneous reports of kidney transplant recipients suggest a strong correlation between detection of the CMV-specific effector T-cell response and the risk of posttransplantation CMV infection [18–28].

The present study found that 24% of the recipients had no detectable cellular immunity to CMV in the QuantiFERON-CMV assay, despite positive pretransplant serology. This is consistent with other studies that showed that 23–50% of anti-CMV IgG pretransplant seropositive recipients had no functional T-cell response to CMV [18, 29]. In another study, 12% of healthy IgG-seropositive subjects had undetectable cellular immunity, based on both the QuantiFERON-CMV and Enzyme-Linked ImmunoSpot tests, suggesting the inability of certain individuals to recognize the pp65 (ppUL83) stimulus peptide or the presence of the atypical or uncommon HLA haplotypes of these subjects [30]. The high proportion of patients with discordance between CMV cellular immunity and the humoral response may also be potentially caused by high susceptibility of the mitogen-dependent response to the action of immunosuppressive drugs, such as tacrolimus and mycophenolate mofetil.

Up to 37% of recipients who were anti-CMV IgG seronegative at the time of transplantation had a positive QuantiFERON-CMV result. This was not caused by false negatives of the CMV serology test because the repetition of CMV serology 90 days posttransplantation confirmed the negative serostatus of these recipients for IgG. The discrepancy between the results of QuantiFERON-CMV and CMV IgG serology in patients before and after kidney transplantation has been reported by others. The positive agreement (73%) between the QuantiFERON-CMV results and CMV serology in hemodialysis patients is less than the positive agreement of 88–97% that was previously reported in healthy adults [19]. In the same study, 13% of the QuantiFERON-CMV measurements revealed positive test results in seronegative dialysis patients, and 21% of CMV-seronegative hemodialysis patients had positive T-Track CMV results. In kidney transplant recipients, the frequency of detecting CMV-reactive effector T cells in CMV-seronegative transplant recipients has been reported to be up to 30% [20]. One possible explanation is that these recipients might have been appropriately sensitized before transplantation, despite the absence of CMV IgG in their serum. Alternatively, primary CMV-specific effector T-cell responses recover quickly and effectively immediately after transplantation, thus providing sufficient protection and control of CMV replication [20]. Further investigations are needed to address these possibilities in kidney transplant candidates. Notably, analyses that are limited to the serological measurement of specific antibodies may not provide a sufficient assessment of the absolute memory repertoire because it excludes preformed CMV-specific memory B cells that can exist in the absence of detectable circulating anti-CMV IgG antibodies [31, 32].

One possible limitation of the present study was that we did not include three cases of indeterminate QuantiFERON-CMV results. The possible inclusion of patients with indeterminate results has been controversial. According to the manufacturer, indeterminate results are not interpretable. For the purposes of analysis, Manuel et al. classified negative and indeterminate results together as nonreactive [33]. Notably, 37% of the organ transplant recipients in their study were treated with thymoglobulin, which is known to increase the rate of lymphocytopenia and the rate of indeterminate QuantiFERON-CMV results. In the aforementioned study, patients with indeterminate results had the highest risk of developing CMV disease, prompting the authors to suggest that indeterminate results reflect a high net state of immunosuppression. However, this possibility was not supported by other investigators who did not confirm the predictive value of indeterminate results for determining the risk of CMV infection [34]. In the present study, the number of patients with indeterminate results was too small to allow assessment of their risk for posttransplantation CMV infection.

In previous studies, the risk of CMV infection in organ transplant recipients was associated with maintenance immunosuppression and the usage of T-cell-depleting antibodies [7, 8]. We did not observe a correlation between maintenance immunosuppression and the occurrence of CMV infection. A possible explanation for this fact is that the majority (98%) of the patients in the present study received the same protocol (i.e., combination of tacrolimus, steroids, and mycophenolate mofetil or mycophenolate sodium as maintenance immunosuppression). Similar to previous reports, the present study confirmed that induction therapy is a risk factor for postprophylaxis CMV infection, but such a conclusion is limited by the small number of patients who present T-cell depletion [35].

Hypogammaglobulinemia is defined as a low level of any or all of the five classes of immunoglobulins. Although the precise mechanism of the evolution of HGG posttransplantation is unclear, secondary HGG has been found to correlate with various factors, including treatment with glucocorticosteroids and mycophenolate mofetil [36]. Other data suggest that HGG develops more frequently after kidney transplantation in patients who are being treated with mycophenolate mofetil compared with patients who are being treated with azathioprine [37]. One of the most interesting findings of the present study was that at the time of prophylaxis discontinuation, HGG was the strongest predictor of CMV infection. Corales et al. reported the occurrence of CMV infection in five of six heart transplant recipients with severe, profound HGG. Lymphocyte subset analysis suggested a decrease in the number of CD4+ T cells, which are required to stimulate B-cell responses [38]. Surprisingly, very sparse information is available on the association between HGG and viral infections following kidney transplantation. Broeders et al. reported that combined HGG (IgG and IgA or IgG and IgM) was associated with more frequent infectious complications, including CMV infection. Similar to our findings, isolated hypo-IgG, although frequent, has not been identified as a predisposing factor for CMV infection after kidney transplantation [39, 40].

## Conclusions

Despite the relatively small sample size in the present study, the findings have relevant clinical implications. Current clinical protocols use very few predictors of the occurrence of postprophylaxis CMV infection. The present results indicate that negative QuantiFERON-CMV results in recipients who receive antiviral prophylaxis is a strong predictor of the risk of postprophylaxis CMV infection, independent of pretransplant CMV serology.

We also found that induction therapy, lymphocytopenia, and HGG on day 90 posttransplantation were risk factors for postprophylaxis CMV infection.

The negative QuantiFERON-CMV assay results suggest an inadequate cellular response and may be used as an indication for extending antiviral prophylaxis or monitoring CMV DNAemia after prophylaxis withdrawal.

## Abbreviations

AIC: Akaike information criterion
AU: Arbitrary unit
CMV: Cytomegalovirus
CV: Coefficient of variation
ELISPOT: The enzyme-linked immunospot
GLM: Generalized linear models
HGG: Hypogammaglobulinemia
IFN-γ: Interferon-γ
IU: International unit
KTx: Kidney transplantation
MDRD: Modification of Diet in Renal Disease
mTor-i: Mammalian target of rapamycin inhibitor
OR: Odds ratio
PHA: Phytohemagglutinin
QF: Quantiferon
QNAT: Quantitative nucleic acid amplification test
RCF: Relative centrifugal force
SAS: Statistical analysis system
VGCV: Valganciclovir
WHO: World Health Organization
